# An Adaptive Sampling Algorithm with Dynamic Iterative Probability Adjustment Incorporating Positional Information

**DOI:** 10.3390/e26060451

**Published:** 2024-05-26

**Authors:** Yanbing Liu, Liping Chen, Yu Chen, Jianwan Ding

**Affiliations:** School of Mechanical Science & Engineering, Huazhong University of Science and Technology, Wuhan 430070, China; d202180319@hust.edu.cn (Y.L.); chenlp@hust.edu.cn (L.C.); d202180318@hust.edu.cn (Y.C.)

**Keywords:** physics-informed neural networks, adaptive sampling algorithm, Dual Inverse Distance Weighting, partial differential equations

## Abstract

Physics-informed neural networks (PINNs) have garnered widespread use for solving a variety of complex partial differential equations (PDEs). Nevertheless, when addressing certain specific problem types, traditional sampling algorithms still reveal deficiencies in efficiency and precision. In response, this paper builds upon the progress of adaptive sampling techniques, addressing the inadequacy of existing algorithms to fully leverage the spatial location information of sample points, and introduces an innovative adaptive sampling method. This approach incorporates the Dual Inverse Distance Weighting (DIDW) algorithm, embedding the spatial characteristics of sampling points within the probability sampling process. Furthermore, it introduces reward factors derived from reinforcement learning principles to dynamically refine the probability sampling formula. This strategy more effectively captures the essential characteristics of PDEs with each iteration. We utilize sparsely connected networks and have adjusted the sampling process, which has proven to effectively reduce the training time. In numerical experiments on fluid mechanics problems, such as the two-dimensional Burgers’ equation with sharp solutions, pipe flow, flow around a circular cylinder, lid-driven cavity flow, and Kovasznay flow, our proposed adaptive sampling algorithm markedly enhances accuracy over conventional PINN methods, validating the algorithm’s efficacy.

## 1. Introduction

The field of fluid mechanics includes numerous partial differential equations (PDEs) essential for studying fluid dynamics. Computational fluid dynamics (CFD) uses numerical analysis and data structures to analyze and solve fluid flow problems, incorporating various numerical solving methods such as the finite element method [1], finite volume method [2], and spectral methods [3]. Despite significant advancements over recent decades, these methods often face complexities due to mesh division in practical applications. Utilizing the capabilities of neural networks as universal function approximators, they become highly effective for solving complex PDEs [4]. In 2019, Raissi et al. [5] introduced physics-informed neural networks (PINNs), a deep learning-based innovative approach to solving PDEs that, unlike traditional numerical methods, are mesh-free and can avoid the complexities of mesh division. Moreover, unlike other neural network strategies for solving PDEs that typically depend on extensive data samples, the PINN framework directly incorporates physical laws into the training process. This approach allows for training without external data and can accurately predict the physical behavior of fluid motion.

During the training of PINNs, especially when tackling complex systems of PDEs or scenarios with irregular geometric domains, ensuring model solution accuracy often necessitates a large number of training samples. Many researchers currently rely on basic sampling strategies, such as uniform random sampling [6,7] and Latin hypercube sampling [8], when employing PINNs to solve various physical models. These approaches neglect the importance of sampling optimization as a crucial step for enhancing model performance, failing to adequately refine critical model areas that significantly influence solution accuracy. This oversight can result in missing key feature regions during the sampling process, leading to insufficient precision in model solutions. In response, adaptive sampling methods have been developed, which intensify sampling in critical areas, such as solution discontinuity regions or near boundaries—akin to mesh refinement practices in traditional numerical methods. These methods focus on strategically selecting sample points, which can better capture the key characteristics and dynamic changes of the solution, thereby enhancing the efficiency and accuracy of PINN.

Lu et al. [9] introduced DeepXDE, a Python library designed for solving PINNs, and proposed a residual-based adaptive refinement algorithm, RAR. This algorithm aims to enhance the initial training set by incorporating sample points with higher residual values from the solution domain, thereby retraining the PINN’s network parameters to ensure effective training near sharp solution fronts. Jeremy et al. [10] improved the PINN’s loss function by integrating gradient information from the partial differential residuals, in conjunction with the adaptive sampling algorithm RAR, thereby accelerating the model’s convergence speed and achieving comparable accuracy with fewer residual points. Gao et al. [11] innovated the sampling process through active learning, calculating probability values for each residual point and selecting new sample points based on these probabilities, thus refining the model’s learning focus. Several scholars [12,13,14] have proposed adaptive sampling approaches with primary improvements in the selection of probability sampling formulas, enhancing the efficiency and accuracy of PINNs in capturing critical solution features. Additionally, some researchers have explored a domain decomposition approach [15,16], dividing the solution domain into multiple regions and strategically adding sample points in specific areas to significantly enhance PINN accuracy. Furthermore, PENG [17] employed node generation techniques [18], similar to the focus on residual value information in the RAR algorithm, by adding sample points with larger residual values, although this method is currently limited to two-dimensional solution spaces. Gu et al. [19] proposed a self-paced learning framework that assigns higher weights to sample points with larger residuals, thereby facilitating the solution of high-dimensional PDE problems.

Adaptive sampling is a strategic approach employed during the training of PINNs to dynamically select or reallocate sample points, aiming to boost the network’s learning efficiency and accuracy. These methods predominantly leverage residual values, which are the discrepancies between the network’s predictions and actual system behaviors, as indicators for redistributing samples. Focusing on areas with high residual values enables the network to intensify learning in regions that are traditionally challenging to model, thus elevating the overall model performance. However, relying exclusively on residual values may prove insufficient for guiding sample selection within complex physical systems. Incorporating positional information is critical, as it provides a comprehensive view that captures the spatial intricacies of physical phenomena. This integration refines sampling algorithms, enabling the network to effectively navigate areas with heightened complexity or variability, thus enhancing the model’s predictive capabilities and accuracy.

To tackle the challenges previously outlined, we find inspiration in the Dual Inverse Distance Weighting (DIDW) algorithm [20], traditionally applied in geoscientific contexts. The essence of the DIDW algorithm lies in its ability to integrate the distance between data points (D-D) and from data points to potential points (D-P). Furthermore, it employs locally variable exponents to dynamically adjust the weights of these distances. Our adaptation of this algorithm for the computational framework of PINNs allows for an enhanced analysis of distances, not only between data points but also between these points and locations where predictions are sought. Through the application of locally varying exponents, our modified DIDW algorithm dynamically adjusts the weighting of these distances, thereby more accurately reflecting the intricacies of local physical phenomena. This adaptation enables PINNs to more effectively prioritize areas that are critical to enhancing the model’s prediction accuracy, thereby improving both learning efficiency and predictive performance. Moreover, to augment the algorithm’s randomness and accelerate convergence, we integrate principles of reinforcement learning by introducing a private reward factor. This innovative addition dynamically adjusts the probability density function for each iteration of the adaptive sampling algorithm, substantially refining the precision of our solution. Through these enhancements, our approach significantly improves the adaptability and efficacy of PINNs in capturing complex physical processes.

The structure of this paper is organized as follows: Section 2 introduces the foundational algorithm underlying PINNs. In Section 3, we provide an in-depth explanation of the Dual Inverse Distance Weighting—Residual-Based Adaptive Refinement (DIDW-RAR) algorithm, detailing its mechanisms and innovations, and introduce the sparsely connected network structure used in PINNs. Section 4 is dedicated to validating our model through its application to a variety of fluid dynamics problems, demonstrating its effectiveness and versatility. We draw our conclusions in the final section, summarizing the key findings and contributions of our work. Additionally, we have included a list of abbreviations and nomenclature in the appendix to facilitate reading.

## 2. Methods

PINNs combine deep learning with physics to solve complex problems in science and engineering. PINNs use the power of neural networks to approximate functions and include physical equations as constraints in the loss function. This approach ensures that PINNs not only learn from data but also obey physical laws. Originating from Raissi’s work [4], PINNs are designed to work with partial differential equations as follows:

(1) $$\begin{array}{l} u_{t}+N_{x}[u]=0,x\in \Omega ,t\in [0,T],\\ u(x,0)=I(x),x\in \Omega ,\\ u(x,t)=B(x,t),x\in \partial \Omega ,t\in [0,T], \end{array}$$

where u(x,t) represents the latent variables that need to be determined, where x and t denote the spatial and temporal coordinates, respectively. The term $u_{t}$ refers to the derivative of u with respect to time, and $N_{x}[\cdot ]$ indicates the differential operator in the equation. The interior and boundary of the solution domain are represented by Ω and ∂Ω, respectively. I(x) and Β(x,t) denote the initial and boundary conditions of the partial differential equation, respectively. Through computation with PINNs, the approximated value of the latent variable u(x,t) can be obtained as $\hat{u}(x,t;\theta )$, where θ are the neural network parameters.

In constructing PINNs, fully connected neural networks (FCNNs) are instrumental in capturing complex nonlinear relationships through dense inter-layer connections. This capability is especially critical for approximating solutions to PDEs. An FCNN consists of multiple hierarchical layers, including an input layer, several hidden layers, and an output layer. In an FCNN, the output of each layer is produced by first calculating a linear combination of the previous layer’s outputs using weight matrices and bias vectors and then applying a nonlinear activation function to this linear combination. Assuming the weight matrix and bias vector for the l-th layer of the neural network are $W_{l}\in R^{N_{l}\times N_{l-1}}$ and $b_{l}\in R^{N_{l}}$, respectively, the output relationship between adjacent layers can be expressed as follows:

(2) $$O^{l}\left(X\right)=\phi^{l}\left(W_{l}O^{l-1}\left(X\right)+b_{l}\right),1\leq l\leq L-1$$

Here, $O^{l}\left(X\right)$ represents the output at the l-th layer of the neural network, where X denotes the input data. We denote the trainable parameters of the neural network by $\theta ={\left\{W_{l},b_{l}\right\}}_{l=1}^{L}$, and ϕ(·) represents the activation function, commonly the hyperbolic tangent function (tanh), in PINNs. Through the aforementioned neural network, an approximate solution to the equation, $\hat{u}(x,t;\theta )$, can be obtained. This approximation is then substituted into the partial differential equation to construct the residual form as follows:

(3) $$f\left(x,t;\theta \right)≔\frac{\partial }{\partial t}\hat{u}\left(x,t;\theta \right)+N_{x}\left[\hat{u}\left(x,t;\theta \right)\right]$$

PINNs compute all partial derivatives and differential operators through automatic differentiation (AD) in PyTorch [21]. The goal is to optimize θ so that the approximated solution $\hat{u}(x,t;\theta )$ adheres to the specified boundary and initial conditions, while also fulfilling the physical constraints dictated by the PDEs. Accordingly, the PINNs’ loss function is divided into three main components: constraints from boundary conditions, constraints from initial conditions, and constraints derived directly from the PDEs themselves. The specific expression for the loss function is as follows:

(4) $$Loss\left(\theta ;N\right)≔\lambda_{f}{MSE}_{PDE}\left(\theta ;N_{f}\right)+\lambda_{b}{MSE}_{BC}\left(\theta ;N_{b}\right)+\lambda_{i}{MSE}_{IC}\left(\theta ;N_{i}\right)$$

The parameters $\lambda_{f}$, $\lambda_{b}$, and $\lambda_{i}$ represent the weighting coefficients for the different loss terms, where ${MSE}_{PDE}(\theta ;N_{f})$, ${MSE}_{BC}(\theta ;N_{b})$, and ${MSE}_{IC}(\theta ;N_{i})$ are defined as follows:

(5) $$\begin{array}{c} {MSE}_{PDE}\left(\theta ;N_{f}\right)=\frac{1}{\left|N_{f}\right|}\sum_{k=1}^{N_{f}}\;∥\frac{\partial \hat{u}\left(x_{f}^{k},t;\theta \right)}{\partial t}+N_{x}\left[\hat{u}\left(x_{f}^{k},t_{f}^{k};\theta \right)\right]{∥}^{2},\\ {MSE}_{BC}\left(\theta ;N_{b}\right)=\frac{1}{\left|N_{b}\right|}\sum_{k=1}^{N_{b}}\;∥B\left(x_{b}^{k},t_{b}^{k};\theta \right){∥}^{2},\\ {MSE}_{IC}\left(\theta ;N_{i}\right)=\frac{1}{\left|N_{i}\right|}\sum_{k=1}^{N_{i}}\;∥I\left(x_{i}^{k},0;\theta \right){∥}^{2}, \end{array}$$

The sets ${\left\{x_{f}^{k},t_{f}^{k}\right\}}_{k=1}^{N_{f}}$, ${\left\{x_{b}^{k},t_{b}^{k}\right\}}_{k=1}^{N_{b}}$, and ${\left\{x_{i}^{k}\right\}}_{k=1}^{N_{i}}$ represent the sets of residual, boundary, and initial training points within the solution domain, respectively, with their corresponding counts being $N_{f}$, $N_{b}$, and $N_{i}$. To minimize the loss function $Loss\left(\theta ;N\right)$, we employ gradient descent algorithms like Adam [22], SGD [23], or L-BFGS [24], aiming to reduce the loss as much as possible towards zero. PINNs utilize the robust capabilities of FCNNs for physics-driven learning, enabling the prediction of physical processes even without direct data, showcasing their significant advantage in handling complex physical scenarios.

**Figure 1:**
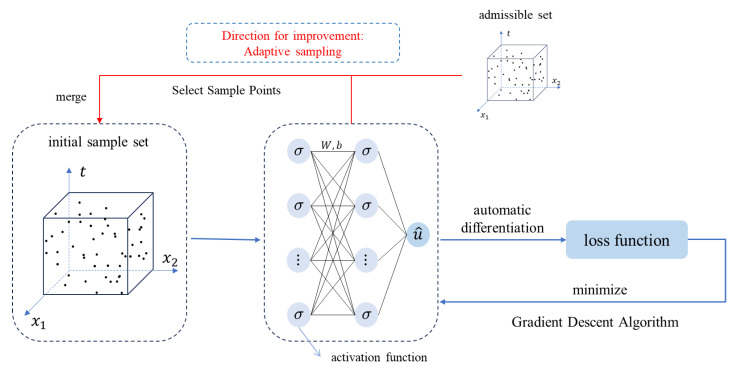
Schematic of PINN in an adaptive sampling algorithm.

Nonetheless, applying FCNNs within PINNs necessitates meticulous design and fine-tuning of the network architecture, including the choice of activation functions and training strategies, to maintain the model’s accuracy and numerical stability. This process involves optimizing the network’s depth and width and carefully balancing the weights among different residuals in the loss function to ensure adherence to the physical laws. Figure 1 presents a flowchart of the PINN framework. The initial training set is processed through an FCNN to obtain a latent solution, which is then differentiated using AD to construct the loss function.

The figure also showcases one research direction in PINNs, namely the adaptive sampling algorithm. The implementation process is described as follows: as shown by the red line in Figure 1, the process begins with a PINN trained on an initial sample set. Subsequently, the admissible sample set is solved using this PINN, and sample points with larger errors are selected for addition. These additional sample points are then combined with the original initial sample set for retraining the PINN.

## 3. Methodology

The adaptive sampling algorithms of PINNs have significantly evolved. In PINNs, the brief flow of the adaptive sampling algorithm is shown in Figure 1.

Initially, the residual-based adaptive refinement with greed (RAR-G) method aimed at increasing sampling points in areas with large residuals from PDEs, using a greedy algorithm to iteratively optimize the distribution of these points. As the technology evolved, the residual-based adaptive refinement with distribution (RAR-D) method was proposed, introducing a probability density function (PDF) related to the residuals. This strategy not only improved the distribution of sampling points but also enhanced the performance and accuracy of PINNs in solving complex PDE problems. Nonetheless, both RAR-G and RAR-D methods primarily concentrated on the magnitude of residuals without fully accounting for the spatial distribution of sampling points. This oversight could lead to ignoring the spatial heterogeneity inherent in physical problems, namely the differing impacts that various spatial regions have on the PDE solutions.

### 3.1. Dual Inverse Distance Weighting Method

Building on the foundations laid by these sampling algorithms, we propose a novel strategy that incorporates the spatial information of sampling points. This ensures that the sampling distribution reflects not only the magnitude of the residuals but also the spatial characteristics of the solution. Incorporating spatial information is crucial for guiding the neural network more effectively in learning the solutions to PDEs, especially for problems with complex boundary conditions or those exhibiting rapid changes in certain areas.

Firstly, we need to compute the corresponding residual value $\varepsilon \left(x,t\right)=\left|f(x,t;\theta )\right|$ for every point within the solution domain. After considering the spatial information of the sampling points, we modify the definition of the probability density function as follows:

(6) $$w\left(x,t\right)\propto \left(\beta \cdot \varepsilon \left(x,t\right)+\gamma \cdot g\left(x,t\right)\right)$$

where $\varepsilon \left(x,t\right)$ denotes the physical property indicator, derived from the residual values, whereas $g\left(x,t\right)$ represents the spatial location indicator. β and γ are non-negative tuning coefficients that balance the importance of the physical property indicators and the spatial location indicators.

We select the DIDW method for the spatial location indicator. This method incorporates local variability into the weight calculation, adjusting the impact of distances between data points and between data points and the potential points via locally adaptive exponents. Such adaptability allows the weights to more accurately reflect the spatial heterogeneity of the studied phenomenon, offering nuanced control over sampling point selection based on their spatial context and the physical property distribution within the region. The implementation process unfolds as follows:

Step 1. Define the Distance Metric

D-P Distance (Data Point to Potential Point Distance): For each potential sampling point (x,t) and points $\left(x_{i},t_{i}\right)$ within the existing sample set S, calculate the distance $d_{i}$ between them.

D-D Distance (Data Point to Data Point Distance): For every pair of sampling points $\left(x_{i},t_{i}\right)$ and $\left(x_{j},t_{j}\right)$ within the existing sample set S, calculate the distance $d_{ij}$ between them.

Step 2. Introduce Locally Varying Exponents

Define two locally varying exponents $p_{1}(x,t)$ and $p_{2}(x,t)$, corresponding to the weight impacts of D-P distance and D-D distance, respectively. These exponents are dynamically adjusted based on the local attributes of the estimation position (x,t) to better adapt to the local characteristics of spatial data. Specifically, $p_{1}(x,t)$ is proportional to the gradient magnitude of the solution, i.e., $p_{1}\left(x,t\right)=\left|{\hat{u}}_{x}(x,t;\theta )\right|$, and $p_{2}(x,t)$ is proportional to the rate of change of the solution’s gradient, i.e., $p_{2}\left(x,t\right)=\left|{\hat{u}}_{xx}(x,t;\theta )\right|$.

Step 3. Calculate Weights

Utilize the DIDW formula to calculate the weight for each potential sampling point:

(7) $$g\left(x,t\right)=\frac{d_{i}^{-p_{1}\left(x,t\right)}\sum_{j=1}^{n}\;d_{ij}^{p_{2}\left(x,t\right)}}{\sum_{i=1}^{n}\;\left[d_{i}^{-p_{1}\left(x,t\right)}\sum_{j=1}^{n}\;d_{ij}^{p_{2}\left(x,t\right)}\right]}$$

This formula takes into account both the distance from potential sampling points to existing sampling points and the distance between sampling points, adjusting their impact through locally varying exponents. We utilize the K-means clustering algorithm [25] for selecting potential sampling points, renowned for its simplicity and rapid execution speed. K-means clustering is performed based on the residual value of each sample point within the admissible set, thereby determining the set of potential sample points.

### 3.2. Dynamically Adjusting the Probability Density Function

Using the same probability density function formula for sampling in every iteration can decelerate the convergence speed of the neural network. To enhance the algorithm’s global optimization capabilities, randomness is strategically incorporated into the selection process for new sampling points. Each time a set of admissible points is formed, the normal sampling process is chosen with a higher probability, while the selection of the admissible point set in a completely random manner is assigned a lower probability. The strategy formula is as follows:

(8) $$\left.x_{i}\left(k\right)=\left\{\begin{array}{cc} norm\left(\left(x_{s},t_{s}\right),e^{1-\beta \left(k-1\right)}\right), & \xi <\varepsilon \\ random\;in\left[\left(x_{min},t_{min}\right),\left(x_{max},t_{max}\right)\right], & \xi \geq \varepsilon \end{array}\right.\right.$$

In the formula, norm() generates sample points based on a normal distribution, where the sampling points selected in the previous round $\left(x_{s},t_{s}\right)$ serve as the mean, and $e^{1-\beta (k-1)}$ determines the variance. This setup implies that the larger the residual value of the sampling points selected in the previous round, the smaller the variance becomes. Consequently, there is a higher probability of selecting the expected value. Furthermore, ξ represents a random number between 0 and 1, and ε is a factor set manually, typically set to ε=0.8. This configuration indicates that the algorithm is 80% likely to employ normal sampling for generating new admissible domains and has a 20% chance of randomly creating admissible domains directly within the solution domain.

Inspired by the principles of reinforcement learning, we establish a reward–penalty factor β(k), which is determined based on the current value of the loss function and its recorded historical values. This factor dictates the update mechanism for the probability density function corresponding to each sample point in the current round, thereby influencing the neural network’s learning rate and convergence outcomes. Within a sampling iteration round, all sample points in the acceptable domain share the same reward factor. Assuming the current training round is k, the reward–penalty function is demonstrated as follows:

(9) $$\beta \left(k\right)=min\left(max\left(0,\frac{L_{mean}-L\left(k\right)}{L_{mean}-L_{min}}\right),1\right)$$

where $L_{mean}$ is the average loss function value of the previous s(s<k) rounds, L(k) is the current value of the loss function, and $L_{min}$ is the historical minimum value of the loss function. From this, it is evident that the value of β(k) is in the range of [0,1], depending on the relationship among $L_{mean}$, $L_{min}$, and L(k), specifically as follows:

(1) $L(k)\geq L_{mean}$: In this case, the currently selected sample points increase the average loss function value of the recent s rounds, indicating a poorly performing sample set. Consequently, the value of β(k) will be 0.
(2) $L_{mean}>L(k)>L_{min}$: Here, the currently selected sample set decreases the average loss function value of the recent s rounds but does not surpass the historical optimum. This indicates a relatively well-performing sample set. As per the formula, the value of β(k) should be in the range of (0, 1). The closer L(k) is to $L_{min}$, the larger the value of β(k) is.
(3) $L(k)\leq L_{min}$: In this scenario, the currently selected parameters outperform the historical optimum, indicating a very well-performing parameter set. According to the formula, the value of β(k) will be 1.

However, this reward mechanism has inherent flaws. Sharing a single reward factor across the entire admissible set leads to a significant issue: For regions within the solution domain that exhibit larger errors, their contribution to reducing the overall loss function is substantial, resulting in a lower target loss function value for the current round. Consequently, the reward factor will be relatively high. On the other hand, other regions in the solution domain that have been selected with fewer sample points exert minimal influence on the target loss function for the current round. This causes the algorithm to continue rewarding these less effective sample points.

To tackle the issue mentioned, this paper introduces a “private reward factor” mechanism, wherein each parameter is assigned its own distinct reward factor. In this refined approach, upon identifying the admissible set, a common reward factor γ is derived using the formula previously discussed. Subsequently, the comprehensive residual value of each sampling point within the admissible set is calculated independently for both the current and previous iteration of the neural network. This serves as a criterion to gauge the performance improvement or decline of individual sampling points. For instance, the formula for assessing the i-th sampling point is presented as follows:

(10) $${err}_{k}\left(x_{i},t_{i}\right)=w_{k-1}\left(x_{i},t_{i}\right)-w_{k}\left(x_{i},t_{i}\right)$$

In the formula, $w_{k-1}(x,t)$ denotes the comprehensive residual value within the admissible set for the PINN at round k−1, and $w_{k}(x,t)$ signifies the comprehensive residual value in the admissible set for the PINN at round k. Observation reveals that a positive deviation, $err_{i}(k)$, signifies a beneficial adjustment of the sampling point in the current round, leading the output of the sigmoid function towards one. On the contrary, a negative deviation suggests an unfavorable selection of the sampling point, driving the sigmoid function’s output towards zero. Outputs of the sigmoid function for other cases will fall in the range of (0, 1). This output value from the sigmoid function is defined as the private reward factor for each sampling point, formulated as

(11) $$\alpha_{k}\left(x_{i},t_{i}\right)=sigmoid\left(C\cdot err_{k}\left(x_{i},t_{i}\right)\right)$$

where C serves as a control factor that can be adjusted to either amplify or diminish the impact of individual sample deviations on the reward factor. The final reward factor for each parameter is obtained by multiplying the private reward factor by the common reward factor, as shown in the following equation:

(12) $$\beta_{k}\left(x_{i},t_{i}\right)=\alpha_{k}\left(x_{i},t_{i}\right)\cdot \gamma$$

In practice, the private reward factor functions as a corrective coefficient for the original reward factor, ensuring that the algorithm does not indiscriminately reward poorly performing sample points simply because of overall satisfactory performance.

With each sample point assigned a unique reward factor, it can then adjust its own probability density function based on the designated update strategy. This strategy determines whether and how to modify the probability density associated with each sample point in the admissible set for the current round, effectively influencing the chance of that parameter being chosen in future learning iterations. The formula for this strategy is outlined as follows:

(13) $$\left.f_{k}\left(x_{i},t_{i}\right)=\right.\alpha_{k}\left(w_{k}\left(x_{i},t_{i}\right)+\beta_{k}\left(x_{i},t_{i}\right)H\left(\left(x_{s},t_{s}\right),\left(x_{i},t_{i}\right)\right)\right),\left(x_{i},t_{i}\right)\in H$$

The formula provided uses the sample point $\left(x_{i},t_{i}\right)$ within the admissible set H as an illustration. In the formula, $\beta_{k}(x_{i},t_{i})$ represents the reward factor associated with the sample point $\left(x_{i},t_{i}\right)$ in the k-th round. Furthermore, $H\left(\left(x_{s},t_{s}),(x_{i},t_{i}\right)\right)$ denotes the Gaussian neighborhood function:

(14) $$H\left(\left(x_{s},t_{s}\right),\left(x_{i},t_{i}\right)\right)=\sum_{j=1}^{n\left(\left(x_{s},t_{s}\right)\right)}{\lambda e}^{-\frac{{\left(\left(x_{s},t_{s}\right)-\left(x_{i},t_{i}\right)\right)}^{2}}{2\sigma^{2}}}$$

Here, $\left(x_{s},t_{s}\right)$ symbolizes the newly added sample points selected in the previous round, while $n((x_{s},t_{s}))$ indicates the number of new sample points added in the previous round. λ and σ represent the amplitude and variance of the Gaussian distribution function, respectively. These can be described as follows:

(15) $$\begin{array}{c} \lambda =\frac{g_{h}}{{\left(x_{i},t_{i}\right)}_{max}-{\left(x_{i},t_{i}\right)}_{min}}\\ \;\\ \sigma =g_{w}\left({\left(x_{i},t_{i}\right)}_{max}-{\left(x_{i},t_{i}\right)}_{min}\right) \end{array}$$

Here, $g_{h}$ and $g_{w}$ are parameters designed to control the amplitude and variance of the Gaussian distribution function, respectively, thus affecting the learning rate of the algorithm. In this paper, we set $g_{h}=g_{w}=1$. Additionally, $\alpha_{k}$ serves as a normalization factor and is defined as follows:

(16) $$\alpha_{k}=\frac{1}{\sum_{H}w_{k}\left(x_{i},t_{i}\right)+\beta_{k}\left(x_{i},t_{i}\right)H\left(\left(x_{s},t_{s}\right),\left(x_{i},t_{i}\right)\right)}$$

Based on the content discussed, the complete process of the DIDW-RAR algorithm (Algorithm 1) can be summarized as follows:

**Algorithm 1.** DIDW-RAR algorithm

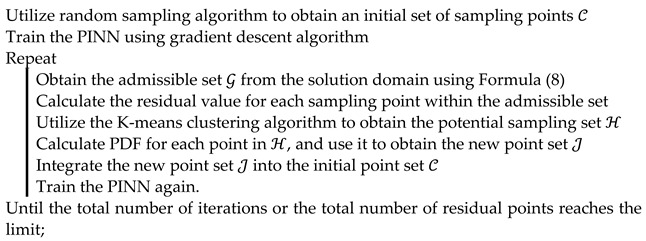



### 3.3. Sparsely Connected Neural Network

Considering that the adaptive sampling algorithm requires multiple trainings of the neural network, and fully connected networks, due to their large number of parameters, occupy significant storage space and considerably increase training time, we have opted to improve PINNs using a sparsely connected neural network.

**Figure 2:**
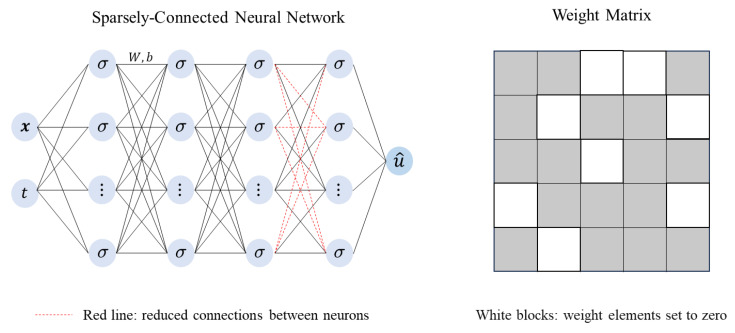
The implementation process of the sparsely connection neural network.

The specific implementation process is shown in Figure 2. In our network, different parts employ varying densities of connections. The initial layers use a fully connected design to capture as many features and complexities from the input data as possible, which is crucial during the early stages of the model because these stages require the formation of sufficient abstraction levels for effective learning. In subsequent layers, we implemented sparse connections by applying a masking operation on the weight matrix to reduce the number of connections between neurons. Specifically, during weight initialization, a random mask is generated based on a predefined level of sparsity and applied to the weight matrix, setting a certain proportion of weight elements to zero. This design maintains sparsity throughout the training process while significantly reducing storage and computational demands. The network and the process of implementing sparsity are illustrated in the diagram.

By reducing the number of connections, the sparse network significantly reduces the multiply–add operations during forward and backward propagation, thus lowering the computational resources required for each training iteration, increasing training speed, and reducing energy consumption. Additionally, sparse connections also decrease the total number of model parameters, directly reducing the memory space required for storing the parameters.

## 4. Results

For this paper, to validate the effectiveness of the proposed adaptive sampling algorithm DIDW-RAR in solving partial differential equations, we conducted a series of numerical experiments. The $L_{2}$ error metric has been selected due to its ability to quantify the deviation between the neural network’s predicted output and the true solution. It allows us to gauge how closely the neural network approximates the solution of the partial differential equation throughout the learning process. The formula for the relative $L_{2}$ error is as follows:

(17) $$L_{2}\;error=\frac{\sqrt{\sum_{i=1}^{N}{\left\|u\left(x_{i},t_{i}\right)-\hat{u}\left(x_{i},t_{i}\right)\right\|}^{2}}}{\sqrt{\sum_{i=1}^{N}{\left\|u\left(x_{i},t_{i}\right)\right\|}^{2}}}$$

Here, $u\left(x_{i},t_{i}\right)$ denotes the reference value of the sample point, while $\hat{u}\left(x_{i},t_{i}\right)$ signifies the exact value of the sample point, with N representing the total number of sample points.

We select several typical cases, including the two-dimensional Burgers’ equation with sharp solutions, and the Navier–Stokes equations under various scenarios such as pipe flow, flow around a circular cylinder, lid-driven cavity flow, and Kovasznay flow. The following sections will provide a detailed description of the solving process for each case.

### 4.1. Burgers’ Equation

Burgers’ equation is a nonlinear partial differential equation that models the behavior of shock waves, commonly encountered in fluid mechanics. It is frequently used to model turbulence, the dynamics of viscous fluids, and a variety of wave phenomena. For our initial investigation, we consider the two-dimensional viscous Burgers’ equation, which is articulated as follows [26]:

(18) $$\left\{\begin{array}{c} u_{t}+uu_{x_{1}}+uu_{x_{2}}=\nu (u_{x_{1}x_{1}}+u_{x_{2}x_{2}}),(x_{1},x_{2},t)\in (-10,10{)}^{2}\times (0,10),\\ u=g,(x_{1},x_{2},t)\in \partial (-10,10{)}^{2}\times (0,10),\\ u=h,(x_{1},x_{2},t)\in (-10,10{)}^{2}\times \{0\}, \end{array}\right.$$

In this paper, we investigate the Burgers’ equation with a diffusion coefficient of v=0.1. Starting from the exact solution $u\left(x_{1},x_{2},t\right)={\left(1+exp(\left(x_{1}+x_{2}-2vt\right)/2v)\right)}^{-1}$, we establish the initial and boundary conditions. Our objective is to approximate the solution across the entire spatiotemporal domain. For this purpose, we construct a deep neural network composed of five hidden layers, each containing 50 neurons, and select the hyperbolic tangent function (tanh) as the activation function. For the distribution of the test set, we have chosen uniformly distributed grid data of 1000 × 1000 at time snapshots *t* = 2.5 and 5, used to compute the relative L2 error of the prediction results.

At the initial stage of training, we employ a uniformly random sampling algorithm to select 1000 boundary training points, 500 initial training points, and 5000 internal training points from the solution domain. Existing adaptive sampling algorithms choose a fixed number of sample points in each sampling round. To reduce the training time of the model, we adjust the cyclic sampling process. In our approach, we conduct 50 rounds of sampling. In the first 20 rounds, we select 50 sample points per round and perform 4000 optimization steps using the Adam optimizer after each selection. In the subsequent 30 rounds, we choose 150 sample points per round. Considering that the Adam optimizer can quickly find a reasonable solution space and the L-BFGS optimizer offers more stable convergence properties, we conduct 2000 steps of Adam optimization followed by 2000 steps of L-BFGS optimization after selecting the sample points.

**Figure 3:**
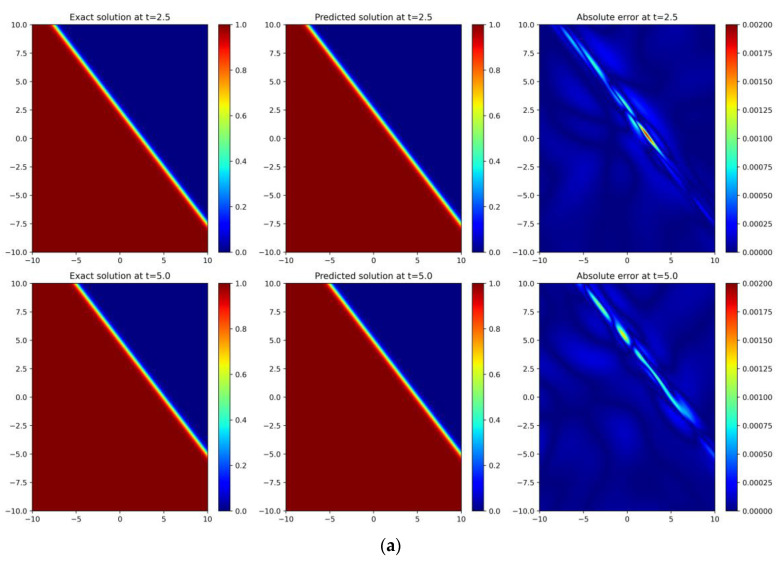

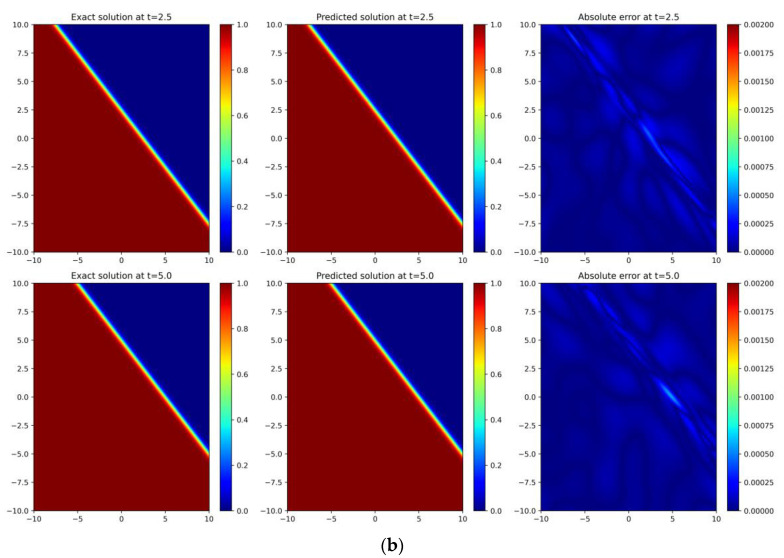
Burgers’ equation: reference velocity, predicted velocity, and absolute point-wise error at *t* = 2.5 and *t* = 5.0. (**a**) Latin hypercube sampling algorithm; (**b**) DIDW-RAR sampling algorithm.

In the experiments conducted for this paper, we utilize the adaptive sampling algorithm; we propose and compare its performance against that of the LHS sampling algorithm. The corresponding fitting results of the two algorithms are shown in Figure 3. At time snapshot *t* = 2.5, the relative L2 error of the DIDW-RAR sampling algorithm is 0.0234%. At time snapshot *t* = 5, the relative L2 error is 0.0316%. If we use the adaptive sampling algorithm to improve solution accuracy, it will require a long runtime. Therefore, we have made modifications to the network structure and the iteration process to minimize the runtime as much as possible. By using sparsely connected neural networks and modifying the cyclic sampling process, the computation time is reduced by 27%.

### 4.2. Navier–Stokes Equation

In the realm of fluid mechanics, the Navier–Stokes equations are of paramount importance as they encapsulate the movement of fluid substances and elucidate the core principles of fluid dynamics. Various fluid phenomena—ranging from advection and flow around a cylinder to flow in a square cavity—represent distinct instances of the N-S equations applied within their unique contexts.

For incompressible Navier–Stokes equations, the dimensionless form of the governing equations is as follows:

(19) $$\left\{\begin{array}{l} u_{t}+(u\cdot \nabla )u=-\nabla p+\frac{1}{Re}\Delta u+f,\\ \nabla \cdot u=0, \end{array}\right.$$

where ∇ represents the Nabla operator, $u=(u_{1},u_{2})$ is the velocity vector, p represents the pressure of the fluid, f denotes a given source term, and Re stands for the Reynolds number.

#### 4.2.1. Pipe Flow

**Figure 4:**
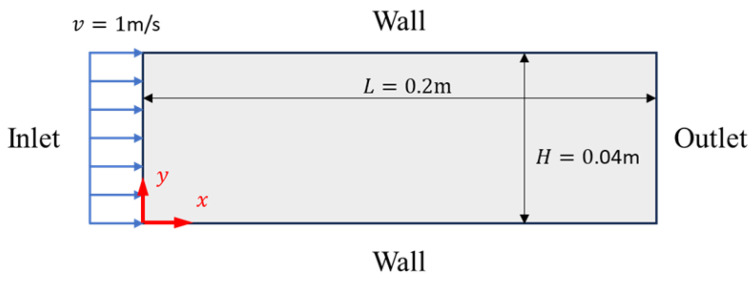
Computational model diagram for pipe flow.

In fluid mechanics research, pipe flow refers to the natural state of fluid movement in the absence of fixed obstacles, which is of particular significance in the fields of meteorology and oceanography, for instance, in analyzing patterns of ocean surface waves or atmospheric flows. We employ the proposed adaptive sampling algorithm, in conjunction with PINN, to simulate advection through a cylindrical object. The inlet is characterized by a uniformly distributed velocity profile, and the outlet is set to zero pressure; non-slip conditions are applied along the boundaries of the pipe, as shown in Figure 4.

During the initial training phase, we select 2000 internal sample points and 500 boundary sample points using uniformly random sampling. Firstly, we conduct 15,000 iterations using the Adam optimizer with a learning rate of $10^{-4}$. Subsequently, employing the DIDW-RAR sampling algorithm, we add 10 sample points to the training set in each of the first twenty rounds and continue with 4000 iterations using the Adam optimizer. In the last twenty rounds, we add 30 sample points per round, followed by 2000 iterations of Adam optimization and 2000 iterations of L-BFGS optimization. The LHS sampling algorithm is also utilized for comparison, ensuring its sampling process is consistent with that of the adaptive algorithm. The network configuration comprises a six-layer fully connected neural network with 50 neurons per layer, using tanh as the activation function. To evaluate the error between the prediction and the reference solution, a uniformly distributed grid of 1000 × 1000 points is used.

**Figure 5:**
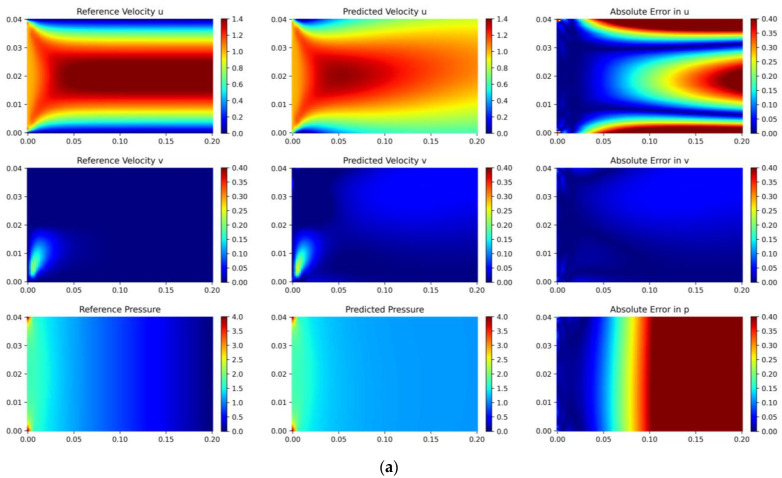

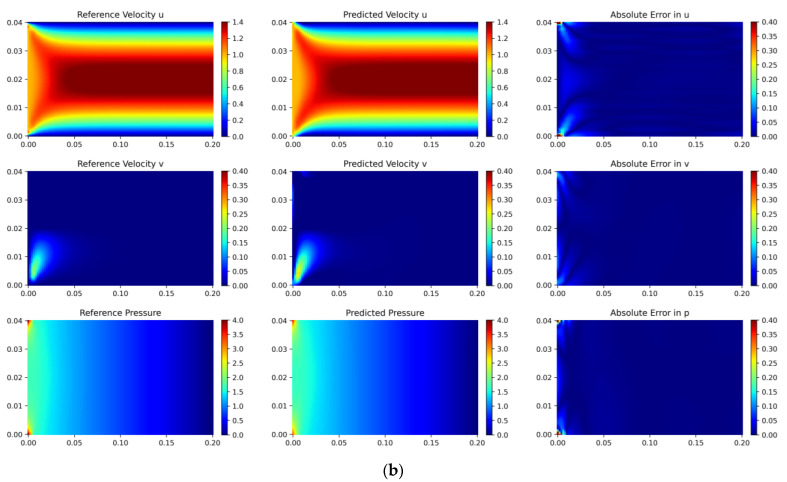
Flow around a pipe flow: reference velocity, predicted velocity, and absolute point-wise error. (**a**) Latin hypercube sampling algorithm; (**b**) DIDW-RAR sampling algorithm.

The fitting results of the two algorithms for the pipe flow are illustrated in Figure 5. The predicted solutions using the LHS algorithm exhibit relative L2 errors of 23.1% for variable *u*, 86% for variable *v*, and 59.2% for variable *p*. In comparison, the predicted solutions derived from the DIDW-RAR algorithm demonstrate relative L2 errors of 2.29% for variable *u*, 3.37% for variable *v*, and 1.96% for variable *p*. Additionally, with our improvements, the training time is reduced by 19%.

#### 4.2.2. Flow around a Circular Cylinder

**Figure 6:**
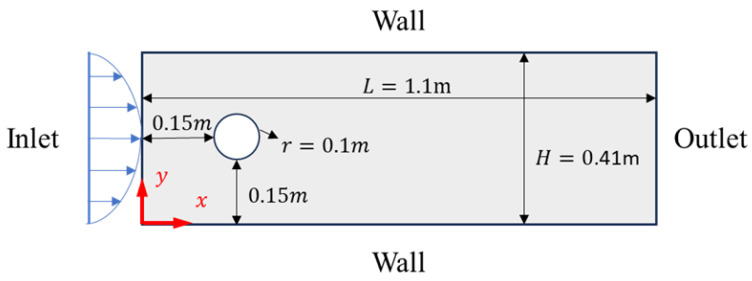
Computational model diagram for flow around a circular cylinder.

Flow around a circular cylinder involves the fluid motion patterns, such as water or air, around a stationary cylindrical body. These flow patterns play a significant role in engineering domains. For example, in the aerospace field, research on flow around a cylinder helps optimize the design of aircraft to reduce drag; in civil engineering, understanding the behavior of fluids flowing around bridge piers is crucial for designing bridges that can withstand wind and waves. Combining PINNs with the proposed adaptive algorithm to solve the laminar and turbulent flow problems around a cylinder, a parabolic velocity distribution is adopted at the inlet, while zero pressure is maintained at the outlet. Non-slip conditions are set at the boundary and inner walls of the cylindrical body, as shown in Figure 6.

In our study, by converting the Navier–Stokes equations into the stream function formulation, we effectively reduce the number of variables required for a solution from three to two. This transformation not only simplifies the problem but also significantly lessens the computational load. Additionally, employing the stream function inherently satisfies the continuity equation for incompressible flows, thereby diminishing the necessity to enforce numerous physical constraints during the solving process. The correlation between the stream function and velocity is established as follows:

(20) $$u=-\frac{\partial \varphi }{\partial y},\;v=-\frac{\partial \varphi }{\partial x}$$

**Figure 7:**
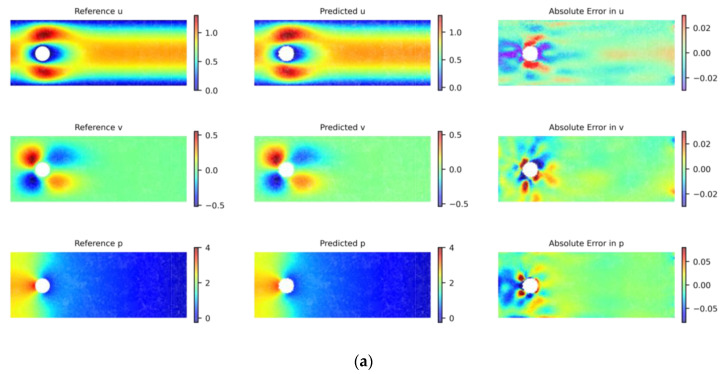

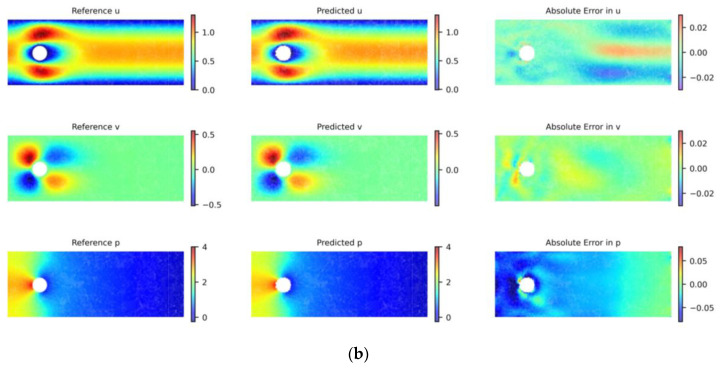
Flow around a circular cylinder: reference velocity, predicted velocity, and absolute point-wise error. (**a**) Latin hypercube sampling algorithm; (**b**) DIDW-RAR sampling algorithm.

We use uniformly random sampling to set 200 sample points at the inlet and outlet, 400 sample points on the upper and lower surfaces of the pipe, 200 sample points on the surface of the cylinder, and 5000 sample points within the solution domain. Initially, the PINN uses the Adam optimizer, starting with a learning rate of $10^{-3}$ for 15,000 iterations and then adjusting the learning rate to $10^{-4}$ for another 15,000 iterations. Subsequently, we employ the adaptive sampling algorithm DIDW-RAR proposed in this paper for one hundred iterations. In the first fifty iterations, we add 25 sample points per iteration and conduct training with 5000 steps using the Adam optimizer. In the last fifty iterations, we add 75 sample points per iteration and conduct training with 2000 steps of Adam optimization followed by 3000 steps of L-BFGS optimization, until a total of 5000 new sample points is added. To ensure consistency and comparability of the experimental results, the process of generating sample points also utilized the LHS algorithm, maintaining consistency with the aforementioned procedure. The relative L2 error is evaluated on a test set consisting of a uniformly distributed grid of 1100 × 410 points. The results of training with different sampling algorithms are shown in Figure 7. Using the LHS algorithm, the L2 errors for the predicted solutions are 1.10% for variable *u*, 3.75% for variable *v*, and 6.66% for variable *p*. For solutions obtained with the DIDW-RAR algorithm, the corresponding L2 errors are 0.54% for variable *u*, 1.32% for variable *v*, and 3.38% for variable *p*. With our improvements to the network and training process, the training time is reduced by 13%.

#### 4.2.3. Lid-Driven Cavity Flow

Square cavity flow pertains to the fluid motion within an enclosure defined by four walls, where typically the top wall is in motion. This seemingly simple model is remarkably capable of demonstrating a vast array of flow phenomena inherent in incompressible fluids, such as vortices, secondary flows, Taylor–Görtler vortices, fluid bifurcation, instabilities, transient flows, and turbulence. Owing to its comprehensive representation of fluid dynamics, square cavity flow is frequently utilized to probe a multitude of theoretical and engineering challenges, finding relevance in fields as diverse as meteorology, navigation, mechanical engineering, and mining. Regarded as a quintessential problem in the study of incompressible fluid flow, our square cavity flow model is designed with a horizontal velocity imparted exclusively at the top surface, while the other three surfaces maintain a zero-velocity condition, which is called lid-driven cavity flow. Non-slip conditions are rigorously applied to all walls. The boundary conditions are delineated as follows:

(21) $$\begin{array}{c} u\left(x,0\right)=0,u\left(x,1\right)=1,u\left(0,y\right)=0,u\left(1,y\right)=0,\\ \;\\ v\left(x,0\right)=0,v\left(x,1\right)=0,v\left(0,y\right)=0,v\left(1,y\right)=0. \end{array}$$

**Figure 8:**
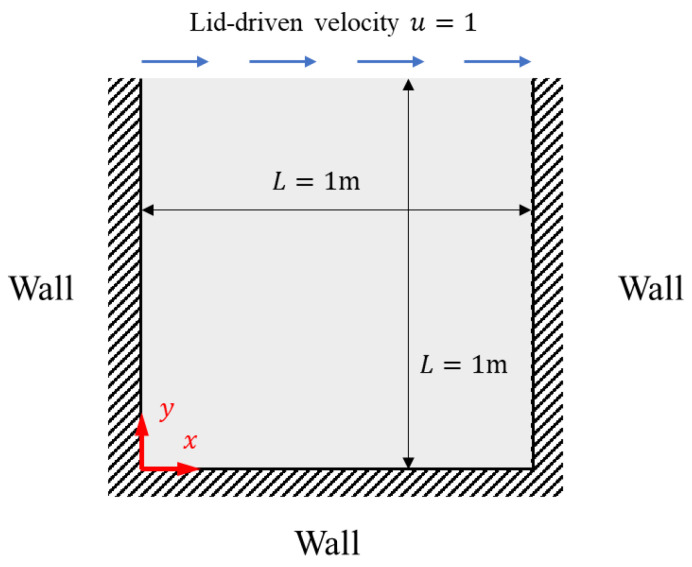
Computational model diagram for lid-driven cavity flow.

The schematic diagram of the lid-driven cavity flow is shown in Figure 8.

To mitigate the influence of boundary conditions on the accuracy of the solution, we integrate lid-driven velocity boundary conditions as hard constraints during the neural network’s training phase. The transformation of these conditions into specific equations is detailed as follows:

(22) $$\begin{array}{c} \bar{u}=xy\left(x-1\right)\hat{u}\\ \;\\ \bar{v}=xy\left(x-1\right)\left(y-1\right)\hat{v}. \end{array}$$

For the implementation of hard constraints with greater ease, our neural network foregoes the computation using the stream function. Within the computational domain, we allocate 5000 initial sample points using uniformly random sampling and begin the training of the PINN with 20,000 Adam optimization steps, followed by 15,000 steps using the L-BFGS method. Subsequently, we employ the adaptive sampling algorithm proposed in this paper for two hundred iterations of training. In the first hundred iterations, we add 20 sample points per iteration and conduct training with 5000 steps using the Adam optimizer. In the following hundred iterations, we add 60 sample points per iteration and conduct training with 2000 steps of Adam followed by 3000 steps of L-BFGS, until a total of 8000 new sample points is added throughout the iterative process. For experimental consistency and comparability, we also create 5000 initial sample points within the computational domain using a uniformly random sampling algorithm and conduct 200 iterations. During each iteration, 40 sample points are randomly selected, following the same training procedure as that employed by the adaptive sampling algorithm.

**Figure 9:**
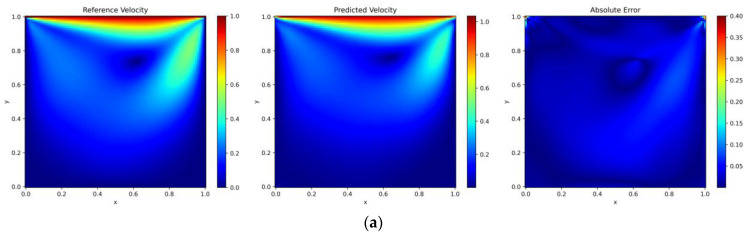

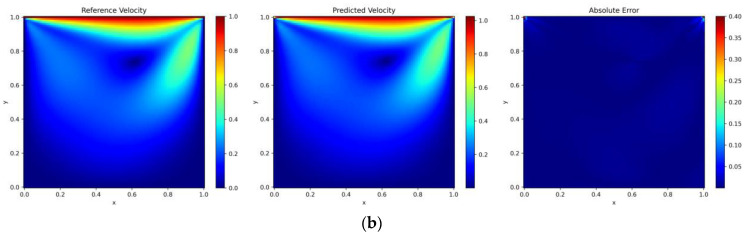
Lid-driven cavity flow: reference velocity, predicted velocity, and absolute point-wise error. (**a**) Latin hypercube sampling algorithm; (**b**) DIDW-RAR sampling algorithm.

We select points from a uniformly distributed 200 × 200 grid as the test set to evaluate the accuracy of the predicted solution. The prediction outcomes from both the random and adaptive sampling algorithms are illustrated in Figure 9. The rightmost section of Figure 9 depicts the point-wise error distribution between the predicted and the reference solutions. The figure demonstrates the high precision of the algorithm introduced in this paper for solving the lid-driven cavity flow problem. When the uniformly random sampling algorithm and the DIDW-RAR sampling algorithm are employed as adaptive sampling algorithms, the relative L2 errors for velocity are 5.4% and 0.91%, respectively. The training time is reduced by 19% compared to the general sampling process.

#### 4.2.4. Kovasznay Flow

**Figure 10:**
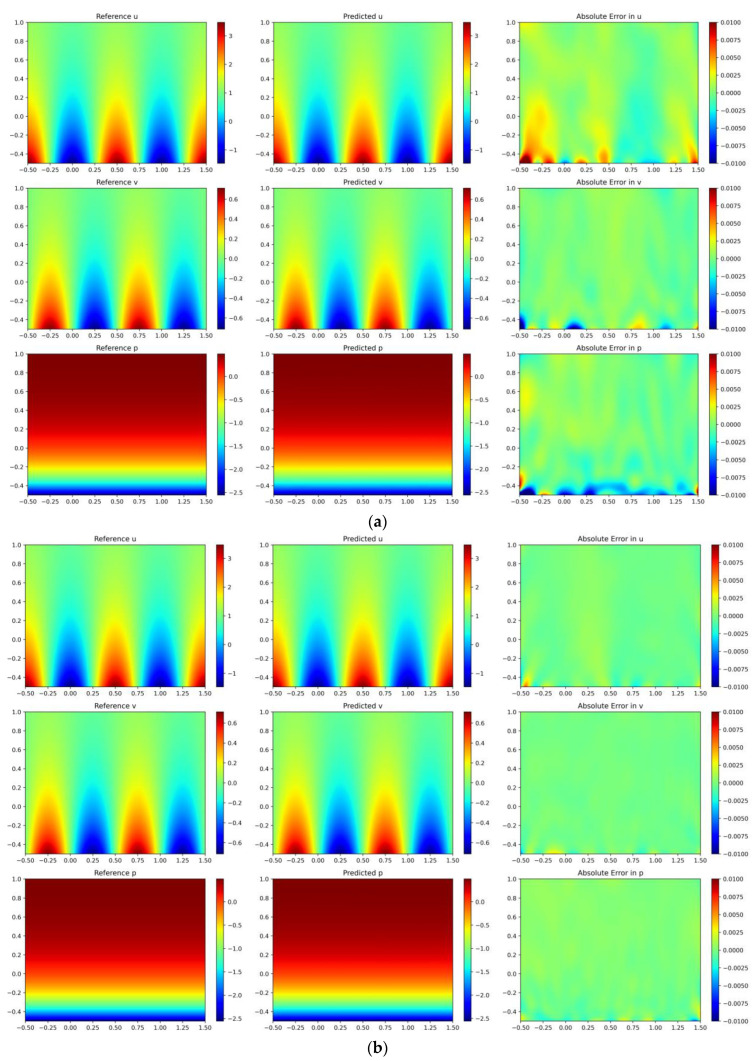
Kovasznay flow: reference velocity, predicted velocity, and absolute point-wise error. (**a**) Latin hypercube sampling algorithm; (**b**) DIDW-RAR sampling algorithm.

Kovasznay flow finds extensive applications in engineering disciplines, particularly in aerodynamics, automotive engineering, the energy sector, and ocean engineering. Research on Kovasznay flow holds significant importance in optimizing designs and improving performance in engineering applications, aiding engineers in better understanding and controlling fluid flow behaviors to enhance system efficiency and performance. We are planning to employ an adaptive sampling algorithm combined with PINN to fit the Kovasznay flow. The domain for solving is set as $\left[-0.5,1\right]\times [-0.5,1.5]$. The analytical expressions for velocity and pressure in the Kovasznay flow are as follows:

(23) $$\begin{array}{l} u(x,y)=1-e^{\lambda x}cos(2\pi y),\\ \nu (x,y)=\frac{\lambda }{2\pi }e^{\lambda x}sin(2\pi y),\\ p(x,y)=\frac{1}{2}\left(1-e^{2\lambda x}\right),\\ where\;\lambda =\frac{1}{2\nu }-\sqrt{\frac{1}{4\nu^{2}}+4\pi^{2}},\nu =\frac{1}{Re}=\frac{1}{20}. \end{array}$$

where $\lambda =1/2\nu -\sqrt{1/4\nu^{2}+4\pi^{2}}$, ν=1/Re=1/20. We use uniformly random sampling to collect 3000 initial sample points within the solution domain and set 500 initial sample points at the boundaries. The training process is consistent with that of the lid-driven cavity flow, with the difference being that we only conduct 100 iterations of sampling. The LHS method is also employed for algorithm comparison. A four-layer fully connected neural network is utilized, featuring 50 neurons per layer, with the tanh function as the activation. We select points from a uniformly distributed 1000 × 1000 grid within the solution domain of [−0.5, 1] × [−0.5, 1.5] as the test set to evaluate the error between the predicted and analytical solutions. The fitting results of the two algorithms are illustrated in Figure 10. For the predicted solutions obtained using the LHS algorithm, the L2 errors are 0.13% for variable *u*, 0.43% for variable *v*, and 0.21% for variable *p*. In contrast, for the solutions derived from the DIDW-RAR algorithm, the L2 errors are 0.04% for variable *u*, 0.12% for variable *v*, and 0.08% for variable *p*. With our improvements to the network and training process, the training time is reduced by 17%.

## 5. Conclusions

In this paper, we propose an adaptive sampling algorithm for PINNs that synergistically combines the DIDW algorithm with the attributes of sampling points. This approach allows for the selection of sample points in accordance with the specific characteristics of the physical problems to be solved and the spatial distribution of the data. For the first time, we incorporate the concept of reinforcement learning into the adaptive sampling algorithm by designing a private reward factor for sampling points based on the error during the iterative process. This innovation modifies the probability sampling formula in each iteration, introducing randomness to the sampling point selection process and enhancing the global optimization capabilities of the algorithm. This not only aids in improving the PINN model’s ability to solve complex physical problems but also effectively increases the model’s learning efficiency and prediction accuracy, especially in cases of sparse data or uneven distribution. Moreover, randomness is introduced into the algorithm, influencing the selection of point generation algorithms in each iteration. To reduce the training time of the model, we optimized the network structure and improved the sampling process, measures that have been proven to effectively reduce training time by 10% to 20%.

We have applied the proposed adaptive sampling algorithm, DIDW-RAR, to solve multiple fluid dynamics problems, including the two-dimensional Burgers’ equation characterized by sharp solutions and various scenarios of the Navier–Stokes equations, such as pipe flow, flow around a circular cylinder, lid-driven cavity flow, and Kovasznay flow. The results demonstrate that with the aid of the proposed adaptive sampling algorithm, PINNs can effectively enhance the accuracy of solutions for the aforementioned problems.

While the PINN model for partial differential equations presented in this paper features a loss function with multiple distinct components, our research has not yet explored the optimal weight balance among these components. It is apparent that the allocation of weights within the loss function directly influences the network’s learning efficacy and peak performance. Consequently, our future work will focus on introducing adaptive weight strategies to precisely calibrate the appropriate weights for each term in the loss function. Additionally, the existing adaptive sampling algorithms largely depend on the iterative addition of new sampling points to enhance learning efficiency and predictive accuracy, a process that necessitates a substantial number of iterations and, therefore, incurs a relatively high computational cost. Although we have implemented a sparse network and designed a specialized sampling process, the reduced runtime is still significantly longer than that of the standard sampling algorithm. To boost efficiency and curtail the required iterations, we aim to investigate novel strategies for more effective increases in sampling points, such as devising entirely new mechanisms for generating sampling points. By implementing these approaches, we aspire to significantly diminish the duration of model training while preserving accuracy, which will also be a pivotal aspect of our continued research.

## Data Availability

Data available on request due to restrictions, e.g., privacy or ethical. The data presented in this study are available on request from the corresponding author. The data are not publicly available due to the Chinese law.

## Abbreviations

The following abbreviations and nomenclature are used in this manuscript:

PINN	Physics-Informed Neural Network
CFD	Computational Fluid Dynamics
PDE	Partial Differential Equation
DIDW	Dual Inverse Distance Weighting
D-D	Distance Between Data Points
D-P	Distance From Data Point to Potential Point
DIDW-RAR	Dual Inverse Distance Weighting—Residual-Based Adaptive Refinement
FCNN	Fully Connected Neural Networks
AD	Automatic Differentiation
PDF	Probability Density Function
RAR-G	Residual-Based Adaptive Refinement with Greed
RAR-D	Residual-Based Adaptive Refinement with Distribution
LHS	Latin Hypercube Sampling
